# Advancing Personalized Care for Neurogenic Bladder: The Case for Continent Urinary Diversion

**DOI:** 10.1002/nau.70347

**Published:** 2026-07-06

**Authors:** Francis A. Jefferson, Maude E. Carmel

**Affiliations:** ^1^ Department of Urology University of Texas Southwestern Medical Center Dallas Texas USA

## Abstract

**Introduction:**

Neurogenic lower urinary tract dysfunction (NLUTD) with a poorly compliant bladder that is refractory to minimally invasive therapies (MIT) represents an “end‐stage” phenotype in which the principal objectives are durable upper urinary tract protection and restoration of continence with acceptable long‐term morbidity.

For most surgically eligible patients in this setting, continent urinary diversion—most commonly a continent cutaneous catheterizable reservoir (e.g., Indiana pouch) and, in highly selected cases, an orthotopic continent diversion—offers a uniquely definitive strategy.

**Methods:**

We conducted a literature review to better characterize the role of continent urinary diversion in the management of refractory NLUTD.

**Results:**

Continent diversion creates a low‐pressure, high‐capacity urinary reservoir while bypassing and often removing the hostile native bladder and dysfunctional outlet, thereby reducing ongoing pelvic urinary morbidity and the surveillance dilemmas inherent to augmentation enterocystoplasty. Compared with an ileal conduit, continent diversion prioritizes continence without an external appliance, an outcome strongly aligned with independence, body image, and sexual well‐being in many neuro‐urological patients.

**Conclusion:**

Although complication and revision rates are meaningful and the need for lifelong follow‐up remains, contemporary long‐term series support durability, preserved renal function, and high satisfaction when patient selection and structured surveillance are optimized.

## 1 Introduction

Neurogenic lower urinary tract dysfunction (NLUTD) includes storage and voiding dysfunction attributable to neurologic disease or injury, and standardized terminology emphasizes urodynamic characterization to identify risk and guide escalation of care [1]. In this context, a poorly compliant bladder and sustained high storage pressures are strongly associated with upper urinary tract injury and progressive renal deterioration in classic neuro‐urological cohorts, supporting “pressure control” as a primary surgical endpoint [2].

When antimuscarinics/β3‐agonists, intradetrusor botulinum toxin, and other MIT fail to establish safe storage pressures, escalation to major reconstruction or diversion is indicated to preserve renal function and reduce infectious morbidity [3, 4]. The operative decision then becomes which definitive endpoint most reliably achieves: (i) durable low‐pressure storage, (ii) continence compatible with long‐term independence, and (iii) an acceptable complication profile over decades of survivorship typical of many neuro‐urological populations. Herein, we present an argument for the utilization of continent urinary diversion in appropriately selected NLUTD patients with a poorly compliant bladder that is refractory to MIT.

### 1.1 The Case for Continent Urinary Diversion

The 2024 European Association of Urology (EAU) guidelines on neurourology emphasize that management should be customized to individual needs and involve a multidisciplinary approach, with lifelong monitoring highlighting the importance of continuous care for neurourological patients [3]. The 2021 American Urological Association (AUA)/Society of Urodynamics, Female Pelvic Medicine Urogenital Reconstruction (SUFU) guidelines state that clinicians should offer urinary diversion to NLUTD patients in whom other options have failed or are inappropriate, in order to improve long‐term quality of life, and note that both incontinent and continent urinary diversion are options for end‐stage bladder or urethral dysfunction [4]. A continent diversion aims to create a low‐pressure, high‐capacity reservoir emptied by intermittent catheterization, thereby aligning the reconstructive anatomy with the principal objective of renal protection while also delivering appliance‐free continence.

In most NLUTD candidates, the dominant choice for continent diversion is a continent cutaneous catheterizable reservoir (e.g., Indiana pouch or ileal reservoir with a catheterizable channel), because urethral emptying and outlet reliability are frequently compromised by neurogenic sphincter dysfunction, impaired sensation, or limited mobility. Orthotopic continent diversion can be considered only in highly selected NLUTD patients with a stable outlet, reliable emptying strategy, and the capacity to self‐catheterize if retention occurs; in routine NLUTD practice, this subset is uncommon [5].

Evidence in adult neuro‐urological populations supports that catheterizable continent stomas are usually functional and continent at follow‐up, although complications are common. A systematic review of continent catheterizable tubes/stomas in adult neuro‐urological patients found that most included studies reported catheterization feasibility rates ≥84% at last follow‐up, and stomal continence typically exceeded 75% [6]. While study quality was limited by retrospective designs and heterogeneity, these data support that a continent catheterizable interface is achievable in adult neuro‐urological cohorts when selection and technique are appropriate.

Very long‐term outcomes are essential in NLUTD. In a contemporary very long‐term cohort of Indiana pouch reservoirs with median follow‐up of 258 months (range 24–452 months), revisions were frequent but durability was demonstrated, with preserved renal function and no pouch malignancies detected [7]. The estimated mean revision‐free survival was 198 months. Reported complete continence rates across continent reservoir cohorts commonly fall in the 75%–85% range, reinforcing the central functional advantage over incontinent diversion [7, 8].

One must also acknowledge the trade‐offs of continent diversions, because these frequently drive long‐term resource use and patient experience. Continent diversion concentrates morbidity in a distinct set of late problems: catheterizable channel stenosis or leakage, reservoir stones, uretero‐reservoir strictures, and metabolic consequences of intestinal urinary storage (e.g., acidosis; vitamin B12 deficiency depending on bowel segment and extent) [4, 6, 7]. In the very long‐term Indiana pouch cohort, two‐thirds of patients underwent surgical revision, illustrating that “dry” often comes with a meaningful likelihood of later reintervention [7]. These realities must be framed as part of the treatment selection process rather than as unexpected failures. Continent diversion is therefore best suited to patients (or reliable caregivers) who can commit to scheduled catheterization, recognize warning symptoms, and participate in lifelong structured follow‐up [4, 6, 7]. When those conditions are met, continent diversion offers a direct, durable pathway to both renal safety and continence‐centered quality of life.

### 1.2 Comparative Considerations Versus Ileal Conduit and Bladder Augmentation

Ileal conduit diversion reliably achieves low‐pressure urinary drainage, but it also requires continuous diversion to an appliance, a trade‐off that can be disproportionately consequential in neuro‐urological patients managing wheelchair seating, transfers, hand function, and skin integrity. In a prospective NLUTD cohort undergoing cystectomy with ileal conduit, urinary‐specific quality of life improved, but generic quality of life did not significantly change, suggesting that conduit diversion can reduce urinary burden without necessarily translating to broader health‐related quality of life gains [9]. In larger neurogenic cohorts, perioperative and long‐term complication rates remain substantial. A spinal cord injury series reported perioperative morbidity in 43% of patients and late complications in 35%, including ureteral anastomosis stenosis, pyelonephritis, and stone disease [10]. Similar patterns are observed in multiple sclerosis cohorts, with meaningful early and late complication rates [11]. These data support conduit diversion as effective and sometimes appropriate, but also underscore that it exchanges upper tract risk for lifelong appliance dependency and a non‐trivial burden of complications [9, 10, 11]. In contrast, the defining benefit of continent diversion is the same renal endpoint with an anatomic solution designed for appliance‐free continence. Importantly, in a large spinal cord injury cohort comparing bladder management strategies, surgery (including augmentation and continent diversion) was associated with fewer bladder symptoms and improved satisfaction compared to clean intermittent catheterization alone [12].

Augmentation enterocystoplasty can achieve excellent urodynamic improvements in NLUTD and remains a major reconstructive option; however, its long‐latency risks are central in young neuro‐urological patients. Reviews emphasize that augmentation is associated with recurrent stone disease, metabolic complications, mucus‐related issues, and a clinically important risk of bladder perforation, which can be catastrophic [13]. Long‐term neurogenic cohorts demonstrate that even with functional improvements, complication rates remain high over time. In adult spina bifida patients with very long follow‐up after augmentation (median 13.6 years), the overall complication rate was 57% and reoperation rate was 14%, with bladder perforation and one case of bladder cancer observed [14]. In a modern spina bifida cohort, 10‐year risk of any reoperation after augmentation was 43.9%, with bladder stones occurring in 28.2% and perforation in 9.6% [15]. The malignancy question remains debated, but systematic review evidence supports that malignant transformation after augmentation for benign disease has been reported, often after long latency (mean 19 years) and frequently at the entero‐urinary anastomosis, creating ongoing uncertainty about optimal surveillance strategies [16, 17]. Additionally, mortality analyses in transitional populations emphasize that rare events such as rupture and sepsis can be fatal, reinforcing that augmentation is an operation that demands lifelong adherence and monitoring [18].

These augmentation‐specific considerations matter for the “end‐stage” NLUTD patient with poor compliance refractory to MIT. Augmentation leaves the native bladder as the central reservoir and often requires additional outlet procedures to achieve continence, while also introducing long‐term risks associated with enterocystoplasty and its bowel–urothelial interface [13, 14, 15, 16, 17]. Continent diversion replaces the reservoir entirely, potentially simplifying the continence framework to catheterizable emptying from a low‐pressure reservoir, at the cost of a higher likelihood of channel‐ and reservoir‐specific late revisions. For surgically eligible patients who prioritize dryness without an appliance and can reliably catheterize and attend follow‐up, continent diversion offers the most direct pairing of the physiological goal (low‐pressure storage) with the functional goal (continence).

## 2 Conclusions

For NLUTD patients with a poorly compliant bladder refractory to MIT, the defining clinical need is a definitive low‐pressure storage strategy that protects the upper urinary tract over decades while optimizing patient‐centered function. Current guideline framing supports urinary diversion as an important option when other treatments have failed, with both continent and incontinent approaches available depending on patient factors [3, 4]. Contemporary long‐term data on continent reservoirs (e.g., Indiana pouch) demonstrate meaningful revision rates but also substantial durability, preserved renal function, and favorable continence in many cohorts [7, 8]. In contrast, ileal conduit diversion—while effective—commits patients to lifelong appliance management with substantial perioperative and late morbidity, and augmentation cystoplasty—while urodynamically beneficial—carries long‐latency risks (perforation, malignancy, stones) and surveillance challenges that accumulate in young neuro‐urological patients [9, 10, 11, 12, 13, 14, 15, 16, 17, 18]. For most patients who can commit to catheterization and lifelong follow‐up, continent urinary diversion offers the most compelling balance of renal protection and continence‐centered quality of life and should be prioritized as the preferred definitive surgical endpoint.

## Author Contributions

**Francis A. Jefferson:** conceptualization, methodology, writing. **Maude E. Carmel:** conceptualization, methodology, writing, supervision.

## Funding

The authors have nothing to report.

## Ethics Statement

The authors have nothing to report.

## Consent

The authors have nothing to report.

## Conflicts of Interest

The authors declare no conflicts of interest.

## Data Availability

Data sharing not applicable to this article as no datasets were generated or analyzed during the current study.
